# Properties of *Modestobacter deserti* sp. nov., a Kind of Novel Phosphate-Solubilizing Actinobacteria Inhabited in the Desert Biological Soil Crusts

**DOI:** 10.3389/fmicb.2021.742798

**Published:** 2021-11-05

**Authors:** Zhu-Ming Jiang, Bing-Huo Zhang, Hong-Min Sun, Tao Zhang, Li-Yan Yu, Yu-Qin Zhang

**Affiliations:** ^1^Institute of Medicinal Biotechnology, Chinese Academy of Medical Sciences & Peking Union Medical College, Beijing, China; ^2^College of Life Science, Jiujiang University, Jiujiang, China

**Keywords:** *Modestobacter deserti*, average nucleotide identity, pan-genome, phenotype, genotype, biological soil crusts

## Abstract

Three Gram-stain-positive, aerobic, motile actinobacterial strains designated as CPCC 205119^T^, CPCC 205215, and CPCC 205251 were isolated from different biological soil crust samples collected from Tengger Desert, China. The 16S rRNA gene sequence comparison of these three strains showed they had almost identical 16S rRNA genes, which were closely related to members of the family *Geodermatophilaceae*, with the highest similarities of 96.3–97.3% to the species of *Modestobacter*. In the phylogenetic tree based on 16S rRNA gene sequences, these isolates clustered into a subclade next to the branch containing the species of *Modestobacter lapidis* and *Modestobacter multiseptatus*, within the lineage of the genus *Modestobacter*. The comparative genomic characteristics (values of ANI, dDDH, AAI, and POCP) and the phenotypic properties (morphological, physiological, and chemotaxonomic characteristics) of these isolates readily supported to affiliate them to the genus *Modestobacter* as a single separate species. For which, we proposed that the isolates CPCC 205119^T^, CPCC 205215, and CPCC 205251 represent a novel species of the genus *Modestobacter* as *Modestobacter deserti* sp. nov. CPCC 205119^T^ (=I12A-02624=NBRC 113528^T^=KCTC 49201^T^) is the type strain. The genome of strain CPCC 205119^T^ consisted of one chromosome (4,843,235bp) containing 4,424 coding genes, 48 tRNA genes, five rRNA genes, three other ncRNA genes, and 101 pseudogenes, with G+C content of 74.7%. The whole-genome sequences analysis indicated that this species contained alkaline phosphatase genes (*phoA*/*phoD*), phosphate transport-related genes (*phoU*, *phnC*, *phnD*, *phnE*, *phoB*, *phoH*, *phoP*, *phoR*, *pitH*, *ppk*, *pstA*, *pstB*, *pstC*, and *pstS*), trehalose-phosphate synthase gene (*otsA*), trehalose 6-phosphate phosphatase gene (*otsB*) and other encoding genes for the properties that help the microorganisms to adapt to harsh environmental conditions prevalent in deserts. Strains of this species could solubilize tricalcium phosphate [Ca_3_(PO_4_)_2_] and phytin, assimilate pyrophosphate, thiophosphate, dithiophosphate, phosphoenol pyruvate, 2-deoxy-d-glucose-6-phosphate, and cysteamine-S-phosphate.

## Introduction

Water scarcity, violent temperature fluctuation, strong ultraviolet radiation, and low-substrate supply are the key abiotic stress factors for life in desert environments. Microbiological soil crusts, the primary stage of biological soil crusts, contribute greatly in stabilization of the sandy surface, soil formation, and carbon and nitrogen assimilation, which provide foundation for other organisms to survive. The “National Desert Ecological Reserve” (NDER) located in Shapotou desert region, on the south east edge of the Tengger Desert, south to the Yellow River, in the northwest of China is the first Chinese NDER. This NDER acts as a model for studying the development of biological soil crusts. Previous study (Sun et al., 2015) found that the members of the family *Geodermatophilaceae* (Normand, 2006) were ubiquitous in different types of crusts in Shapotou NDER. Among the validly described species of the family *Geodermatophilaceae*, most validly described type strains, especially the *Modestobacter* (a major genus in the family *Geodermatophilaceae*) members were isolated from harsh environments, including desert soils (Busarakam et al., 2016; Golińska et al., 2020), sandstone (Trujillo et al., 2015), stone surfaces (Normand et al., 2012; Montero-Calasanz et al., 2019), as well as the newly recognized species *Modestobacter excelsi*^1^ from a high altitude Atacama Desert soil (Golinska et al., 2020). The genome study of *Modestobacter* revealed that the multiple copies of genes, such as *coxSML* (carbon monoxide dehydrogenase related genes), *katA* (catalase coding gene), and *uvrACD* (UvrABC system protein coding genes; Chouaia et al., 2012; Gtari et al., 2012; Sghaier et al., 2016) may serve as the genetic basis to help the strains to adapt to extreme ecological niches. Therefore, *Modestobacter* strains from the desert biological soil crusts may be ideal model microorganisms to investigate the microbiological soil crusts development in depth. At the time of writing, the genus *Modestobacter* comprised of 11 validly named species.^1^ Generally, the members of the genus *Modestobacter* are characterized as Gram-staining-positive, aerobic, pink-pigmented, and short rod-shaped actinobacteria, with major polar lipid composition of diphosphatidylglycerol (DPG), phosphatidylglycerol (PG), phosphatidylethanolamine (PE), phosphatidylmethylethanolamine (PME), phosphatidylinositol (PI), and phosphatidylinositol mannosides (PIM). The major respiratory quinone is MK-9(H_4_), and MK-8(H_4_), MK-9(H_6_), MK-9(H_2_), and MK-10(H_4_) may be present. Major fatty acids are iso-C_16:0_ and iso-C_15:0_. The DNA G+C content ranges from 68 to 74.1%.

The primary goal of the present research was to collect and identify the *Modestobacter* cultures from Shapotou NDER, and study the properties of *Modestobacter* members inhabiting in the biological soil crusts in desert niches. The detailed phenotypic and genotypic properties resulted from these strains could be helpful to systematically demonstrate their ecological adaptation mechanism and their ecological function. As a result, strains CPCC 205119^T^, CPCC 205215, and CPCC 205251 were isolated from biological soil crusts collected from Tengger Desert, showing phosphate-solubilizing activity. Based on the phenotypic and genotypic data, a novel species *Modestobacter deserti* sp. nov. is proposed, with the isolate CPCC 205119^T^ as the type strain.

## Materials and Methods

### Acquisition of Samples

The moss-dominated soil crusts samples designated IMB12100 and IMB12108 were collected from Cuiliu ditch (37°25′38″N, 104°35′9″E, 1,691mH) and Yiwan spring (37°35′50″N, 104°34′59″E, 1,329mH), respectively, and the cyanobacteria-dominated soil crusts sample CCL12125 was from the North-road experimental area (37°25′38″N, 104°35′8″E, 1,340mH), in the middle of Shapotou NDER (36°39′–37°41′N, 104°25′–105°40′E, 1,300–1,700mH). Different samples were sealed in sterilized envelopes following collection and taken to the laboratory within 1week of collection. All samples were immediately processed for the isolation of microorganisms after arriving at the laboratory, and the remaining samples were maintained at −80°C.

### Isolation and Identification of *Modestobacter* Strains

Dilution plating method was employed for the isolation of microorganisms from the sand samples. Approximately 0.2–0.3ml of the 10^−3^ dilution was dropped into isolation agar Petri dishes and spread evenly on the surface. The isolation medium of PYG (gL^−1^; peptone 3, yeast extract 5, glycerol 10, glycine betaine 1.25, sodium pyruvate 1.25, and pH 7.5) and agar (1.5% w/v) was prepared and autoclaved separately, to avoid high H_2_O_2_ concentration in media prepared by autoclaving agar and phosphate buffer together (Tanaka et al., 2014), and then mixed the agar with the nutrient content when cooling to 55°C. Subsequently, supplemented with 0.1% (*v*/*v*) of compound trace salts solution (FeSO_4_·7H_2_O 0.2g, MnCl_2_·2H_2_O 0.1g, ZnSO_4_·7H_2_O 0.1g, sterilized water 100ml), 1% (*v*/*v*) of compound vitamin mixture (vitamin B1 1mg, vitamin B2 1mg, vitamin B3 1mg, vitamin B6 1mg, phenylalanine 1mg, biotin 1mg, alanine 0.3mg, suspended in 100ml sterilized water) and cycloheximide to the final concentration of 50mgL^−1^. Vitamin mixture and cycloheximide were all filter-sterilized.

The Petri dishes spread with soil suspension were incubated at 28°C for 3weeks and distinct colonies were streaked into newly prepared PYG agar dishes to purify the cultures. The purified isolates were maintained on PYG slants at 4°C and also as glycerol suspensions (20%, *v*/*v*) at −80°C.

The *Modestobacter*-like strains were primarily identified according to the 16S rRNA gene sequence comparison following next steps.

Extraction of genomic DNA and PCR amplification of the strain’s 16S rRNA gene were carried out as described by Li et al. (2007). The obtained sequence was compared with available 16S rRNA gene sequences from GenBank using the BLAST program and the EzTaxon-e server^2^ to determine an approximate taxonomic affiliation (Yoon et al., 2017a). Multiple sequence alignment and analysis of the data were performed by using the molecular evolutionary genetics analysis (MEGA) software package version X (Kumar et al., 2018). The phylogenetic trees were reconstructed by the software package MEGA version X using the neighbour-joining (Kimura, 1979) and confirmed by maximum-likelihood (Felsenstein, 1981) and maximum-parsimony (Kluge and Farris, 1969) tree-making methods. Bootstrap analysis with 1,000 replicates was performed to obtain the confidence level of the branches (Felsenstein, 1985).

### Growth Conditions and Morphological Tests

Growth characteristics of the strains were tested using ISP 2 (Shirlng and Gottlieb, 1966), GYM (gL^−1^; glucose 4, yeast extract 4, malt extract 10, calcium carbonate 2, pH 7.5), Tryptic soy agar (TSA, Difco), Reasoner’s 2A agar (R2A, Difco), nutrition agar (NA), and PYG agar. The temperature range for growth was tested at 4, 10, 15, 20, 28, 30, 32, 35, 37, and 40°C using ISP 2 broth for cultivation 2weeks. The pH range (5.0–12.0, at intervals of 1 pH unit) for growth was observed in ISP 2 broth using the buffer system described by Xu et al. (2005). Tolerance to NaCl was examined using GYM broth as the basal medium with different NaCl concentrations [0–10% (*w*/*v*; at 1% intervals)].

Colonies characteristics and pigments production were observed and recorded after 3-day incubation on GYM agar^3^ at 28°C. The Gram reaction was tested by the standard Gram-staining method as described by Magee et al. and observed using light microscopy (BH-2, Olympus). Motility of cells was examined on GYM semi-solid medium agar (0.3%, *w*/*v*) and then checked using light microscopy. The cellular morphology of the test strains was studied using transmission electron microscopy (JEOL JEM-1010) after 6-day incubation on GYM semi-solid slant agar. Before cells were mounted on formvar-coated copper grids (Electron Microscopy Science), they were negatively stained using 2% (*w*/*v*) uranyl acetate for 15s.

### Physiological Tests

The reference strains of *M. lapidis* DSM 100206^T^ and *M. multiseptatus* DSM 44406^T^ obtained from DSMZ were carried out some assays in parallel.

The assimilation of carbon compounds, nitrogen sources, phosphorus, and sulfur sources were tested at 28°C using Biolog GEN III, PM3B, and PM4A Microplates, respectively, in an Omnilog device (BIOLOG Inc., Hayward, CA, United States). Other metabolic characters were determined by API 50CH and API ZYM test kits (bioMerieux) according to the manufacturer’s instructions. Results were evaluated after incubation at 28°C for 72–168h. The oxidase activity was detected using API oxidase reagent (bioMeriéux) according to the manufacturer’s instructions. The catalase activity was determined by observation of bubble production in 3% (*v*/*v*) H_2_O_2_. The abilities of strains to produce H_2_S and indole, hydrolysis of cellulose, gelatin, and starch were examined according to previously described procedures (Zhou et al., 1998; Yuan et al., 2008).

The ability of these three strains and the reference strains *M. lapidis* DSM 100206^T^ and *M. multiseptatus* DSM 44406^T^ to solubilize insoluble phosphate were determined on plates using phosphate-solubilizing media and confirmed using broth culture method (Zhang et al., 2016), with little modification addressed as follows.

Inocula for phosphate solubilization test were prepared from culture grown in GYM broth (28°C, 3days). Cultures were centrifuged (4,860*g*, 20min, 4°C), and the collected biomass re-suspended in small aliquots of sterilized distilled water. Cell suspension, adjusted to approximately 1×10^6^CFU/ml, was inoculated into the phosphate-solubilizing medium (gL^−1^) [glucose, 10; (NH_4_)_2_SO_4_, 0.5; MgSO_4_·7H_2_O, 0.3; NaCl, 0.3; KCl, 0.3; FeSO_4_·4H_2_O, 0.036; MnSO_4_·4H_2_O, 0.03; insoluble phosphorus sources, 2 (including tricalcium phosphate or phytin); distilled water, 1,000ml; pH 7.0]. For the control, the bacterial cell inoculum was replaced with sterile water. Culture broth was centrifuged (4,860*g*, 20min, 4°C) on the 2nd day of incubation, and the amount of available phosphorus in the supernatant determined colorimetrically using standard protocol as described previously (Ministry of Agriculture of the People’s Republic of China, 2010).

Reaction mixture containing 5ml supernatant, 5ml Mo-Sb reagent solution were adjusted to a final volume of 50ml with distilled water, briefly mixed and kept incubated at 20°C for 30min. Absorbance of the reaction mixture was monitored at 700nm against a standard of potassium phosphate. The Mo-Sb reagent solution contained (per liter) sulfuric acid, 2.87mol; ammonium molybdate, 8.1mmol; antimonyl potassium tartrate, 1.5mmol; ascorbic acid, 85.2mmol (added into the solution just before use).

Concentration of the available phosphorus in the culture media was calculated based on the following equation:


$$X=\frac{\left(P\times V1\times K\right)}{V2}$$

where *X* represents available phosphorus in the culture media; *P*, available phosphorus calculated for a reaction mixture; *V*1, total volume of reaction mixture; *V*2 volume of culture supernatant added in the reaction mixture; *K*, the dilution ratio used for measuring the absorbance.

### Chemotaxonomic Tests

Biomass for chemotaxonomic studies of the strains was obtained by cultivation in flasks on a rotary shaker (180rpm) using GYM broth at 28°C for 4days except that cellular fatty acid extraction and analysis were conducted using the cultures harvested from Tryptic soy broth (TSB). The diagnostic isomers of diaminopimelic acid in the whole cell hydrolysates (4N HCl, 100°C, 15h) of these strains were subjected to thin-layer chromatography on cellulose plates using the solvent system of Schleifer and Kandler (1972). The sugar analysis of whole cell hydrolysates followed procedures described by Staneck and Roberts (1974). Polar lipids were extracted and examined by two-dimensional TLC and identified using previously described procedures (Minnikin et al., 1984). Menaquinones were isolated using the method of Collins et al. (1977) and were analysed by HPLC (Groth et al., 1997). Analysis of the whole cell fatty acid pattern followed the described methods using the MIDI system (Microbial ID, Inc., Newark, Del; Kroppenstedt, 1985; Meier et al., 1993). MIDI Sherlock Version 6.0 and ACTIN1 database were employed for this analysis.

### Whole-Genomic Study

#### Genome Sequencing and Assembly

The whole-genome sequencing was implemented using an Illumina HiSeq 4000 system (Illumina, SanDiego, CA, United States) at the Beijing Genomics Institute (Shenzhen, China). Genomic DNA was sheared randomly to construct three read libraries with length of 300bp by a Bioruptor ultrasonicator (Diagenode, Denville, NJ, United States) and physico-chemical methods. The paired-end fragment libraries were sequenced according to the Illumina HiSeq 4000 system’s protocol. Raw reads of low quality from paired-end sequencing (those with consecutive bases covered by fewer than five reads) were discarded. The sequenced reads were assembled using SOAPdenovo v1.05 software.

#### Genome Component Prediction

Gene prediction was performed on the genome assembly by glimmer3^4^ with Hidden Markov models. The tRNAscan-SE (Lowe and Eddy, 1997), RNAmmer, and the Rfam databases were employed for sorting of tRNA, rRNA, and sRNAs, respectively. The tandem repeats annotation was obtained using the Tandem Repeat Finder,^5^ and the minisatellite DNA and microsatellite DNA selected based on the number and length of repeat units. The Genomic Island Suite of Tools (GIST) used for genomicis lands analysis^6^ with IslandPath-DIOMB, SIGI-HMM, IslandPicker method. Prophage regions were predicted using the PHAge Search Tool (PHAST) web server^7^ and CRISPR identification using CRISPRFinder.

### Gene Annotation

Rapid Annotation using Subsystem Technology (RAST; Aziz et al., 2008) was applied for annotation of the assembled whole genomic sequences of the family *Geodermatophilaceae*. The protein sequence of each genome annotated by RAST was used for downstream analysis such as stress-responding genes retrieval, pan-genome analysis, and calculation of AAI and POCP values. For pathway analysis, the predicted proteins sequences were uploaded to KEGG Automatic Annotation Server with “for prokaryotes” and “bidirectional best hit” options. The website of UniProt^8^ was implemented for the validation of stress response genes. Predictions of gene clusters for natural products were performed using antiSMASH (Blin et al., 2019).

### Whole Genome Based Taxonomy

Genomic robust indexes, i.e., ANI (average nucleotide identity), dDDH (digital DNA-DNA hybridisation), AAI (average amino identity), and POCP (percentage of conserved proteins) were calculated for the definition and classification of the novel species of the genus *Modestobacter*. ANI Calculator (Yoon et al., 2017b),^9^ Genome-to-Genome Distance Calculator (Meier-Kolthoff et al., 2013),^10^ Average Amino acid Identity (Rodriguez-R and Konstantinidis, 2016),^11^ and POCP described by Qin et al. (2014) were used to calculate the ANI, dDDH, AAI, and POCP, respectively.

Bacterial Pan-genome Analysis (BPGA) pipeline was applied for analysis of the genomic diversity of the *Modestobacter* members. The protein sequences of strains CPCC 205119^T^, CPCC 205215, and CPCC 205251 were annotated by RAST 2.0. The protein sequences of other *Modestobacter* strains were accessed from NCBI. Pan-genome analysis was performed by BPGA 1.3 using default settings (Chaudhari et al., 2016), and the pan- and core-genome analysis of the *Modestobacter* spp. was also performed by using the Roary pipeline (Page et al., 2015). A total of 22 protein sequence files annotated from the 22 corresponding strains’ whole genome sequences were used to generate orthologous gene/protein clusters (homologous families) by USEARCH clustering tool and construct the phylogenetic tree using the concatenated core genes by BPGA. Except the strain *M. lacusdianchii* JXJ CY 19^T^, without available genome sequence in the database, all other 22 strains were also included in the 16S rRNA gene phylogenetic tree. Each homologous family was given a conserved value (CV) based on the frequency it occurs in the three genomes. Different CV reflects the distribution frequency of the homologous gene in these three strains. The larger CV indicates that the gene is more widely distributed in the strains of the species *M. deserti* sp. nov. and the more conserved the gene family is in the species *M. deserti*. In the pan-genome of the three strains, if the CVs of some homologous families were 3, these homologous families were considered as the part of core genome; those homologous families with the CV of 2 or 1 were regarded as accessory genes or unique genes, respectively. The core, accessory, unique and exclusively absent genes were retrieved using USEARCH clustering tool. BPGA performed the evolutionary analysis based on concatenated core gene alignments and binary (presence/absence) pan-matrix. Gene matrix was calculated using similarity or dissimilarity in contribution of genes to orthologous gene clusters. For core genome based phylogenetic tree, BPGA first extracted the protein sequences (excluding paralogs) from 20 random orthologous gene clusters to generate core genome phylogeny tree. BPGA automated multiple sequences alignments using MUSCLE. All alignments were concatenated and a neighbor-joining phylogenetic tree was constructed.

## Results and Analysis

### Isolation and Identification of *Modestobacter* Strains

Strains designated as CPCC 205119^T^ and CPCC 205215 were isolated from moss-dominated soil crust samples IMB12100 and IMB12108, respectively, and strain CPCC 205251 was acquired from cyanobacteria-dominated soil crust sample CCL12125, collected from Shapotou NDER in Tengger Desert, China.

**Figure 1:**
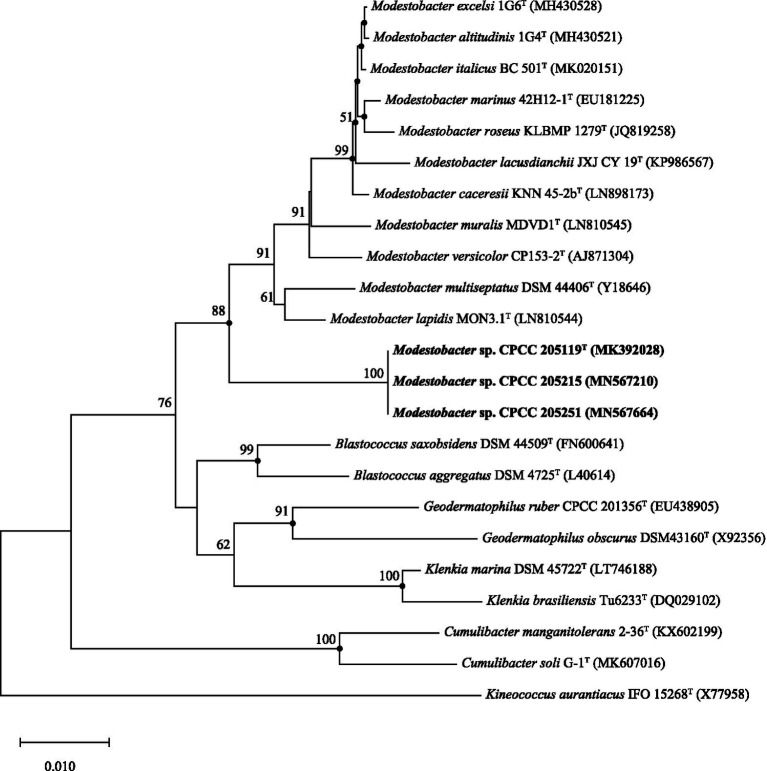
Neighbour-joining tree based on 16S rRNA gene sequences showing the relationship of the strains CPCC 205119^T^, CPCC 205215, and CPCC 205251 with representatives of the family *Geodermatophilaceae*. Filled circles indicate that the corresponding nodes were also recovered in the trees generated with the maximum-likelihood and maximum-parsimony methods. Bootstrap values (>50%) are shown as percentages of 1,000 replicates. Bar, 1nt substitutions per 100nt.

The complete 16S rRNA gene sequences of strains CPCC 205119^T^ (1,529bp), CPCC 205215 (1,529bp), and CPCC 205251 (1,529bp) were obtained. Pairwise alignment indicated that these three newly isolated strains contained almost identical 16S rRNA genes. BLAST search results showed that they exhibited the highest similarities with the validly described species of the genus *Modestobacter*, i.e., *M. lapidis* DSM 100206^T^ (97.3%), *M. multiseptatus* DSM 44406^T^ (97.3%), and other type strains of the genus *Modestobacter* (<97.0%). These similarity values were all lower than 98.65%, which was proposed by Kim et al. as the threshold for differentiating two genic species according to the 16S rRNA gene similarity (Kim et al., 2014). In the Neighbour-Joining phylogenetic tree based on the 16S rRNA gene sequences, strains CPCC 205119^T^, CPCC 205215, and CPCC 205251 formed a stable subclade next to the subclade containing *M. lapidis* DSM 100206^T^ and *M. multiseptatus* DSM 44406^T^ in the genus of *Modestobacter* lineage, within the family *Geodermatophilaceae* (Figure 1), which showed almost the same case in maximum-parsimony, maximum-likelihood trees. These results indicated these three strains could be affiliated to the genus of *Modestobacter* as a distinct species.

### Genome-Based Classification

Assembly sequences for the draft genomes of CPCC 205119^T^, CPCC 205215, and CPCC 205251 were deposited in GenBank with the accession number JAAGWK000000000, WGGQ00000000, and JAABOZ000000000. The detailed genome repertoires were given in the supplementary materials (Supplementary Table S1). In the phylogenetic tree based on the concatenated core genes, strains CPCC 205119^T^, CPCC 205215, and CPCC 205251 clustered in a unique branch in the lineage of the genus *Modestobacter*, with the closest evolutionary distance with the branch formed by *M. lapidis* DSM 100206^T^ and *M. multiseptatus* DSM 44402 (Supplementary Figure S1). The dDDH values between strain CPCC 205119^T^ and their closest phylogenic neighbors ranged in 20.4–22.7%, which was far below than the cut-off value (70%) used to classify bacterial strains of the same species (Auch et al., 2010); the ANI values between strain CPCC 205119^T^ and their closest phylogenic neighbors ranged in 79.5–79.7%, which were also much lower than the threshold for bacterial species delineation (95–96%; Kim et al., 2014); the AAI values between strain CPCC 205119^T^ and their closest phylogenic neighbors ranged in 71.1–71.8%, which were also much lower than the threshold for bacterial species delineation (~ 90%; Rodriguez-R and Konstantinidis, 2014); the POCP values between strain CPCC 205119^T^ and their closest phylogenic neighbors ranged in 53.4–58.5%, which were all higher than threshold for bacterial genus delineation (50%), while lower than the threshold for bacterial species delineation (70%; Qin et al., 2014; Table 1). While the pairwise values of dDDH, ANI and AAI and POCP among these three strains readily confirmed to classify these three strains as one species (Supplementary Table S2). Therefore, the above results strongly supported the proposal that the three strains represent a single novel species (*M. deserti* sp. nov.) of the genus *Modestobacter*. The calculated G+C contents of these strains were in the range of 74.6–74.7%.

**Table 1:** The values of ANI, dDDH, AAI, and POCP of the whole genome DNA assembly sequences between the strain CPCC 205119^T^ and the closest phylogenic neighbours.

	CPCC 205119^T^			
	ANI (%)	dDDH (%)	AAI (%)	POCP (%)
*Modestobacter lapidis* DSM 100206^T^	79.7	22.7	71.8	58.5
*Modestobacter multiseptatus* DSM 44402	79.5	20.4	71.1	53.4

ANI, values of average nucleotide identity; dDDH, digital DNA–DNA hybridization; AAI, average amino acid identity; POCP, percentage of conserved proteins.

### Genes Associated With Stress Response

**Figure 2:**
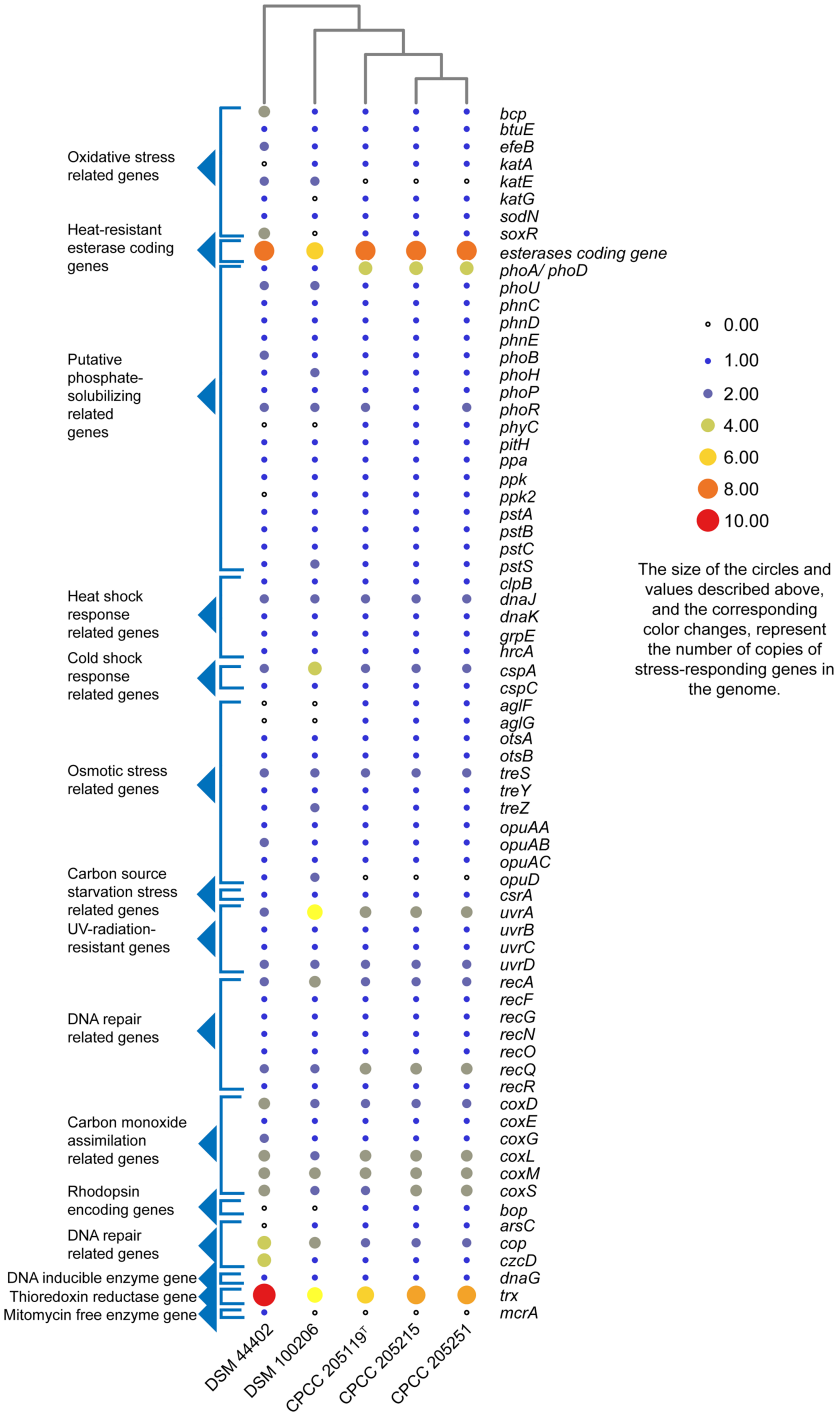
Heatmap of putative stress-responding genes predicted in the genomes of strains CPCC 205119^T^, CPCC 205215, CPCC 205251, and their closest phylogenic neighbors according to the copy number of the genes from the Rapid Annotation using Subsystem Technology (RAST) annotation.

The whole genome of strains CPCC 205119^T^, CPCC 205215, and CPCC 205251 contained 4,480, 4,443, and 4,523 genes, respectively (Supplementary Table S1). The strains *M. lapidis* DSM 100206^T^ isolated from building rock surface and *M. multisptatus* DSM 44402 isolated from Antarctica’s dry and cold Valley composed of 4,366 and 6,094 genes, respectively, which were included as reference. The putative stress-responding related genes were retrieved in the whole genome DNA annotation results (Figure 2). The heat shock response related genes (*clpB*, *dnaK*, *dnaJ*, *grpE*, and *hrcA* gene; Li et al., 2011), the cold shock response related genes (*cspA*, *cspC*; Essoussi et al., 2010), osmotic stress related genes (*aglF, aglG, otsA, otsB, treS, treY, treZ, opuAA, opuAB, opuAC*, and *opuD*; Kempf and Bremer, 1995; Pan et al., 2008; Reina-Bueno et al., 2012), oxidative stress-related genes (*bcp*, *btuE*, *efeB*, *katA*, *katE*, *katG*, *sodN* and *soxR*; Martindale and Holbrook, 2002), carbon source starvation stress-related genes (*csrA*; Romeo et al., 1993; Romeo, 1998), UV-radiation-resistant genes (*uvrA*, *uvrB*, *uvrC*, and *uvrD*; Long et al., 2020), DNA repair related genes (*recA*, *recF*, *recG*, *recN*, *recO*, *recQ*, and *recR*; Hickson, 2003; Manthei et al., 2015), heat-resistant esterase coding genes [esterase (EC 3.1.1.X) coding genes; Essoussi et al., 2010; Jaouani et al., 2012], alkaline phosphatase genes (*phoA*/*phoD*), pyrophosphatase coding gene (*ppa*), and phytase coding gene (*phyC*; Pasamontes et al., 1997), phosphate starvation-inducible protein coding gene (*phoH*), phosphate transport-related genes (*phoU*, *phnC*, *phnD*, *phnE*, *phoB*, *phoH*, *phoP*, *phoR*, *pitH*, *ppk*, *pstA*, *pstB*, *pstC*, and *pstS*; Song et al., 2020), carbon monoxide assimilation-related genes (*coxLSM* gene cluster, *coxD* and *coxE*; Lorite et al., 2000), rhodopsin-encoding genes (*bop*; Shand and Betlach, 1994), arsenate resistance genes (*arsC*; Chouaia et al., 2012), copper resistance related genes (*cop*), various metal resistance genes (*czcD*), DNA inducible enzyme gene (*dnaG*), thioredoxin reductase gene (*trx*), and mitomycin free radical oxidase gene (*mcrA*; Normand et al., 2012) were predicted from all or partial of these strains. A total of 99 copies, 100 copies, 101 copies, 99 copies, and 111 copies of stress response related genes accounted for 2.21, 2.25, 2.23, 2.26, and 1.82%, respectively, in their whole genomes. These data showed stress-responding related genes seemed richer in the microorganisms inhabited in deserts (these three newly isolates) and the rock surface (*M. lapidis* DSM 100206^T^) than that in Antarctica’s Valley (*M. multisptatus* DSM 44402).

### Pan-genome Analysis of the Species *Modestobacter deserti*

**Figure 3:**
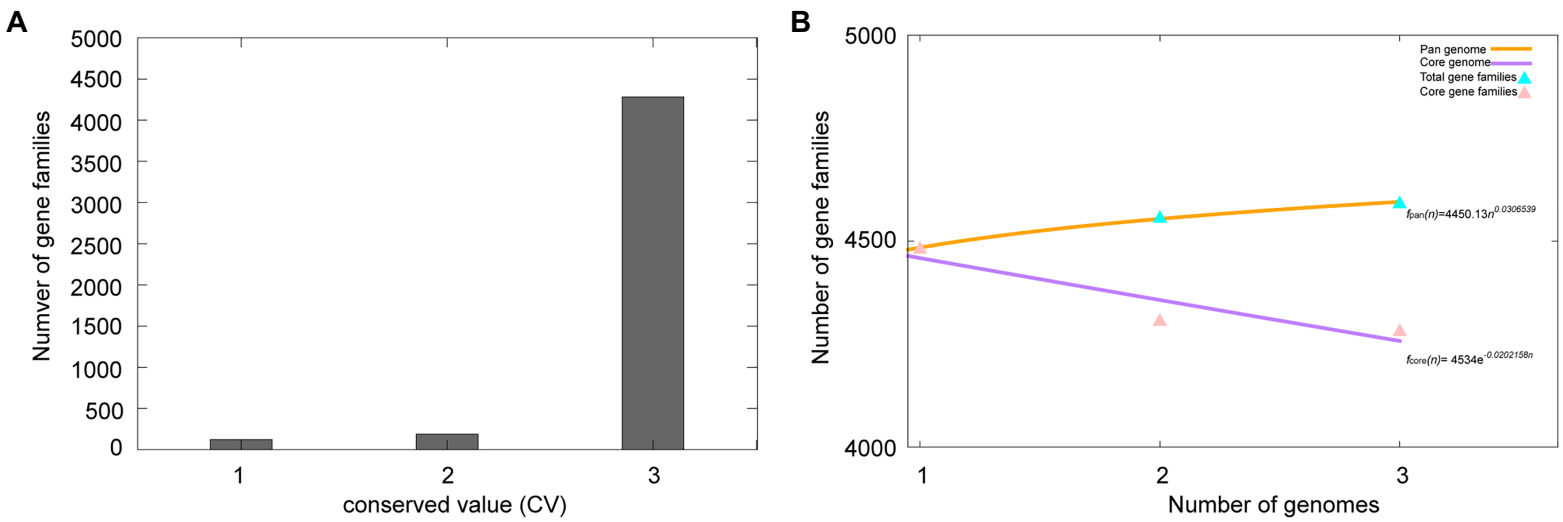
Overview of the pan-genome generated by Bacterial Pan-genome Analysis (BPGA) using three strains of the species *Modestobacter deserti*. **(A)** The gene family frequency spectrum. **(B)** The pan genome profile trends of the species *Modestobacter deserti* obtained using clustering tools USEARCH.

A total of 13,532 protein-coding genes (Table 2) were sorted from the genomes of these three strains of the species *M. deserti*, which were divided into 4,592 homologous families by cluster analysis. Histograms were constructed according to different CVs (Figure 3A). Among them, there were a total of 4,282 core genes commonly shared by these three strains (CV=3), accounting for about 93.2% of the total number of homologous gene families. The accessory genes (188 genes; CV=2) accounted for about 4.1% of the homologous gene families in the newly proposed species. The proportion of the unique genes (122 genes; CV=1) was about 2.7% (Supplementary Figure S2).

**Table 2:** The pan-genome profile of the species *Modestobacter deserti* sp. nov.

Genome number	Organism name	No. of core genes	No. of accessory genes	No. of unique genes	No. of exclusively absent genes
1	*M. deserti* CPCC 205119^T^	4,282	158	42	30
2	*M. deserti* CPCC 205215	4,282	55	45	133
3	*M. deserti* CPCC 205251	4,282	163	35	25

The relationship between the pan-genome size and the number of genomes of the species, and the relationship between the number of core genes and the number of genomes were deduced (Figure 3B) by using all the protein sequences extracted from these three strains of the species *M. deserti*. The functional relationship between pan-genome size (*f*_pan_) and the number of genomes (*n*) was obtained by fitting, as follows:


$$f_{\mathrm{pan}}\left(n\right)=4450.13\times n^{0.0306539}$$

Meanwhile, the functional relationship between the number of core genes (*f*_core_) and the number of genomes (*n*) was obtained by fitting, as follows:


$$f_{\mathrm{core}}\left(n\right)=4534\times e^{-0.0202158n}$$

It could be observed from the pan-genome fitting curve in the Figure 3B that, with the increasing number of sequenced genomes, the pan-genome size tended to a plateau. Accordingly, it could be inferred that the pan-genome of the novel species *M. deserti* was almost closed.

**Figure 4:**
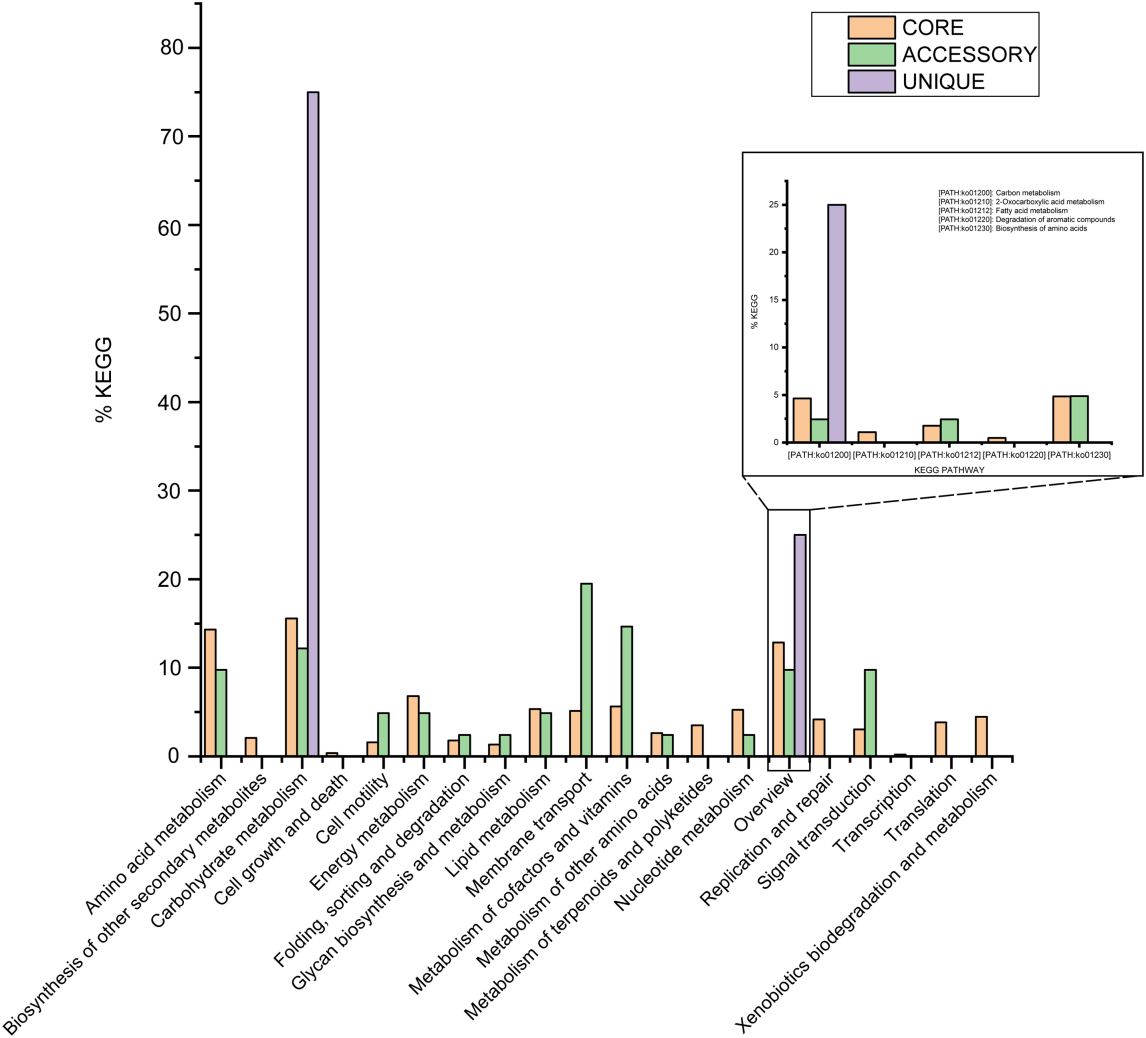
The assigned metabolic pathways associated with the core, accessory, and unique genes among the species *Modestobacter deserti* from the KEGG database.

Out of 4,592 genes (clusters), BPGA could map 2,508 (54.6%) to KEGG (Kyoto Encyclopedia of Genes and Genomes) pathways, i.e., core genes (2,457, 98.0%), accessory genes (47, 1.9%) and unique genes (4, 0.1%). Having filtered some KEGG pathway related to eukaryotes, we obtained an overview on the metabolic pathway corresponding to the gene(s) in the pan-genome of the species *M. deserti*. A large number of core genes (2,305, 15.6%) were involved in carbohydrate metabolism, amino acid metabolism (14.3%), some other elementary metabolism (biosynthesis of amino acids, 4.9%; carbon metabolism, 4.6%; fatty acid metabolism, 1.8%; 2-oxocarboxylic acid metabolism, 1.1% and degradation of aromatic compounds, 0.5%; 12.8%), energy metabolism (6.8%), metabolism of cofactors and vitamins (5.6%), lipid metabolism (5.3%), nucleotide metabolism (5.3%), and membrane transport (5.1%). Accessory and unique genes appeared to be enriched in membrane transport, carbohydrate metabolism, as well as metabolism of cofactors and vitamins. Among the accessory genes (41), the major portion of genes seemed related to membrane transport (19.5%), metabolism of cofactors and vitamins (14.6%), carbohydrate metabolism (12.2%), amino acid metabolism (9.8%), some other elementary metabolism (biosynthesis of amino acids, 4.9%; carbon metabolism, 2.4% and fatty acid metabolism, 2.4%; 9.8%), signal transduction (9.8%), signal transduction (9.8%). Unique genes (4) seemed to be mainly enriched in carbohydrate metabolism (75%), and carbon metabolism (25%; Figure 4).

### Phenotypic Properties

#### Morphological and Physiological Characteristics

**Figure 5:**
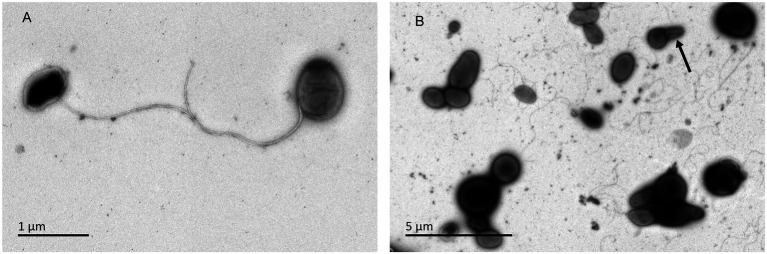
Transmission electron micrograph of strain CPCC 205119^T^ grown on GYM semi-solid medium (0.3% agar) for 7days at 28°C. Bar, 2μm. **(A)** Showing the flagella; **(B)** arrow, indicating the bud-like structure.

Orange to red colored colonies with a maximum diameter of 1.1mm were opaque, convex and irregular circle on ISP 2, GYM, TSA, R_2_A, NA, and PYG agar after incubation for 72h at 28°C. No diffusible pigments were observed on any tested media. Cells of strains CPCC 205119^T^, CPCC 205215, and CPCC 205251 were aerobic, Gram-staining-positive, non-spore-forming, cocci (diameter of 0.5–0.8μm) and/or short rods (0.5–0.6μm in width and 0.8–1.1μm in length), motile with polar flagella. Bud-like structure was observed for some cells (Figure 5). These three strains grew well on ISP 2 agar and GYM agar, moderate growth occurred on and R_2_A agar, TSA, NA and PYG media. Growth of these strains was observed at 10–37°C, pH 6.0–11.0, in ISP 2 medium with the presence of 0–3% (*w*/*v*) NaCl. The optimum growth occurred at 28–32°C, pH 6.0–8.0, with the absence of NaCl. The catalase, oxidase and phosphate solubilizing activity of these strains were positive. None of these strains can hydrolyze gelatin, CM-cellulose (carboxymethyl cellulose), urea and starch. Nitrate was not reduced and H_2_S was not produced.

Detailed physiological and biochemical characteristics of these strains from Biolog GEN III, PM3B, and PM4A Microplates, and API 50CH and API ZYM test kits are given in the description of the species and in the supplementary materials as Supplementary Table S3.

In the liquid culture assay, strains CPCC 205119^T^, CPCC 205215, and CPCC 205251 could solubilize tricalcium phosphate [Ca_3_(PO_4_)_2_] and phytin with a detection of additional available phosphorus of (4.72±0.34) mg/L and (36.78±1.75) mg/L, respectively.

#### Chemotaxonomic Characteristics

These three isolates were found to contain alanine, glutamic acid, glycine, and *meso*-diaminopimelic (*meso*-DAP) in the cell wall. Whole-cell hydrolysates yielded arabinose, glucose and ribose. The cellular polar lipids contained DPG, PE, PG, PI and PIM, PME as well as small amounts of unidentified phospholipid (UPL) and aminophospholipid (APL; Supplementary Figure S3). In the menaquinones extraction, tetrahydrogenated menaquinones with nine isoprene units as the predominant isoprenologue, i.e., MK-9(H_4_) (78.8–82.3% of total menaquinone composition) with minor amounts of MK-8(H_4_) (17.7–21.2%); The cellular fatty acids were C_18:1_*ω*9*c* (18.9–29.7%), iso-C_16:0_ (11.2–20.9%), C_16:0_ (6.7–11.0%), C_17:1_*ω*8*c* (4.2–11.8%), C_16:1_*ω*7*c* (6.6–9.0%), iso-C_15:0_ (6.1–8.0%), C_18:0_ (4.3–5.8%; Supplementary Table S4).

### Taxonomic Study Conclusion

The 16S rRNA gene sequence comparison results and the phylogenic analysis suggest that strains CPCC 205119^T^, CPCC 205215, and CPCC 205251 represent a single novel species of the genus *Modestobacter*. This proposal was well approved by the whole genome research repertoires including ANI, dDDH, AAI, POCP, and the pan-genome phylogenic analysis. The phenotypic properties of strains CPCC 205119^T^, CPCC 205215, and CPCC 205251, such as the assimilation of carbon sources and chemotaxonomic characteristics (Table 3) well supported the classification these three stains as one so far unknown species in the genus *Modestobacter*. Therefore, based on the above phenotypic and genotypic data, we suggested to establish a new species in the genus *Modestobacter*, for which the name *M. deserti* sp. nov. was proposed, with strain CPCC 205119^T^ as the type strain.

**Table 3:** Differentiating phenotypic characteristics of strain CPCC 205119^T^, CPCC 205215, CPCC 205251, and their phylogenetically related species of the genus *Modestobacter*.

Characteristic	1	2	3	4	5
Colony colour	Orange or pink	Orange or pink	Orange or pink	Orange, black or yellow white	Pale pink
Flagella	+	+	+	+	−
*Temperature for growth (°C)*					
Range	10–37	10–37	10–37	20–37	0–28
Optimum	28–30	28–30	28–30	28	19–21
*pH for growth*					
Range	6.0–11.0	6.0–11.0	6.0–11.0	6.0–9.0	4.0–9.5
Optimum	7.0–8.0	7.0–8.0	7.0–8.0	7.5	8.0–8.5
Tolenrance of NaCl (%)	0–3	0–3	0–3	0–8	0–6
Reduction of nitrate	−	−	−	+	+
Urease	+	+	+	+	−
Production of H_2_S	−	−	−	−	+
*Enzyme activities (API ZYM)*					
Acid phosphatase, esterase (C4), esterase lipase (C8), leucine arylamidase, valine arylamidase, naphthol-AS-B1-phosphohydrolase, *β*-glucosidase	+	+	+	+	−
Alkaline phosphatase, lipase (C14), trypsin, *α*-galactosidase, *β*-galactosidase	+	+	+	−	−
*Utilization of (Biolog GEN III)*					
Dextrin	+	+	+	+	−
d-fructose-6-PO_4_, inosine	+	−	−	+	+
d-fucose, l-rhamnose	−	−	−	−	+
d-galactose, d-mannose, d-salicin, *α*-d-lactose	+	+	+	−	+
d-glucose-6-PO_4_	+	−	−	+	−
d-melibiose, gentiobiose	−	−	−	+	−
d-rnaffinose	−	−	−	+	+
Glucuronamide	−	+	−	+	−
*N*-acetyl-d-glucosamine, *N*-acetyl-*β*-d-mannosamine	−	−	−	−	+
d-arabitol, d-mannitol	−	−	−	−	+
d-sorbitol	−	−	−	+	−
Glycerol	+	+	+	−	+
myo-inositol	+	−	+	−	+
d-serine	−	−	−	+	−
l-alanine, l-arginine, l-histidine	−	−	−	−	+
l-glutamic acid	+	+	+	−	+
Gelatin	+	+	+	+	−
Acetoacetic acid	−	+	+	+	+
Bromo-succinic acid, d-malic acid, propionic acid	+	+	+	+	−
Citric acid, d-galacturonic acid, l-pyroglutamic acid, *p*-hydroxy-phenylacetic acid	−	−	−	+	−
d-saccharic acid, l-lactic acid, *β*-Hydroxy-D,l-butyric acid	−	−	−	−	+
d-aspartic acid, mucic acid, quinic acid, *α*-keto-glutaric acid	−	−	−	+	+
d-gluconic acid	+	−	−	−	+
Formic acid	−	+	−	−	−
Methyl pyruvate, *α*-hydroxy-butyric acid	−	+	+	−	−
Pectin	−	+	+	+	−
Tween 40	+	+	+	−	−
DNA G+C content (%)	74.6	74.7	74.7	72	69.9
Phospholipids profile	APL1, APL2, DPG, PE, PG, PI, PIM, PME, UPL	APL1, APL2, DPG, PE, PG, PI, PIM, PME, UPL	APL1, APL2, DPG, PE, PG, PI, PIM, PME, UPL	DPG, PE, PG, PI, PIM	DPG, PE, PG, PI
Major Menaquinone(s)	MK-9(H_4_)	MK-9(H_4_)	MK-9(H_4_)	MK-9(H_4_)	MK-9(H_4_), MK-8(H_4_) and MK-9(H_6_)
Fatty acids components (>5%)	C_18:1_ *ω*9*c*, iso-C_16:0_, C_16:0_, iso- C_15:0_, C_16:1_ *ω*7*c*, C_18:0_	C_18:1_ *ω*9*c*, iso-C_16:0_, C_17:1_ *ω*8*c*, iso- C_15:0_, C_16:1_ *ω*7*c*, C_16:0_	C_18:1_ *ω*9*c*, C_17:1_ *ω*8*c*, iso-C_16:0_, C_16:1_ *ω*7*c*, C_16:0_, iso- C_15:0_, C_18:0_	iso-C_16:0_, iso-C_15:0_, C_16:1_ *ω*9*c*, C_16:0_, C_17:1_ *ω*9*c*	iso-C_16:0_, C_18:1_, anteiso-C_17:0_, cycle-C_19:0_
Isolation source	Cyanobacteria-dominated soil crusts of the Tengger Desert, China	Moss-dominated soil crusts of the Tengger Desert, China	Cyanobacteria-dominated soil crusts of the Tengger Desert, China	Surface of deteriorated sandstone of a historic building, Spain	Soils from Linnaeus Terrace, Antarctica

1, CPCC 205119^T^ (data from this study); 2, CPCC 205215 (data from this study); 3, CPCC 205251 (data from this study); 4, *M. lapidis* DSM 100206^T^ (Trujillo et al., 2015); 5, *M. multiseptatus* DSM 44406^T^ (Golińska et al., 2020). All strains are Gram-reaction-positive, positive for motility, catalase and oxidase; contain meso-diaminopimelic in the cell wall and arabinose, glucose and ribose in the whole-cell hydrolysis. In API ZYM kits, all strains are positive for *α*-chymotrypsin, *α*-glucosidase and cystine arylamidase, negative for *α*-fucosidase, *α*-galactosidase, *α*-Mannosidase, *N*-acetyl-*β*-glucosaminidase and *β*-glucuronidase. In Biolog GEN III (MicroPlate), all strains are positive for acetic acid, *α*-d-glucose, *α*-keto-butyric acid, d-cellobiose, d-fructose, d-maltose, d-trehalose, d-turanose, l-aspartic acid, l-malic acid, and sucrose assimilation; negative for the utilization of 3-methyl glucose, acetoacetic acid, d-glucuronic acid, d-lactic acid methyl ester, d-serine, formic acid, glycyl-l-proline, l-fucose, l-galactonic acid lactone, l-serine, methyl pyruvate, *N*-acetyl-neuraminic acid, *N*-acetyl-d-galactosamine, stachyose, *β*-methyl-d-glucoside and γ-amino-butryric acid. DPG, diphosphatidylglycerol; PE, phosphatidylethanolamine; PME, phosphatidylmethylethanolamine; PG, phosphatidylglycerol; PI, phosphatidylinositol; PIM, phosphatidylinositol mannoside; APL, aminophospholipid; UPL, unidentified phospholipid. +, Positive; −, negative.

### Description of *Modestobacter deserti* sp. nov.

*Modestobacter deserti* sp. nov. (de.ser′ti. L. gen. n. *deserti* of a desert, where the organisms were acquired).

Cells are aerobic, Gram-staining-positive, cocci- to rod-shaped, motile, and non-sporing actinobacterium. The colonies are irregular, opaque, orange-red on most tested media. Grows at 10–37°C and pH 6.0–11.0, with optima at 28–30°C and pH 7.0–8.0, respectively. Grows in NaCl at concentrations up to not more than 3%. The activity of oxidase and catalase is positive. The production of H_2_S, indole and melanin, hydrolysis of gelatin, starch, and cellulose are negative. Cells are positive for acid phosphatase, alkaline phosphatase, cystine arylamidase, esterase lipase (C8), esterase (C4), leucine arylamidase, lipase (C14), naphthol-AS-B1-phosphohydrolase, trypsin, valine arylamidase, *α*-glucosidase, *β*-glucosidase, *β*-galactosidase, and weekly positive for *α*-chymotrypsin, *α*-galactosidase in API ZYM strip. Acetic acid, bromo-succinic acid, d-cellobiose, Dextrin, d-fructose, d-galactose, d-malic acid, d-maltose, d-mannose, d-salicin, d-trehalose, d-turanose, gelatin, glycerol, l-aspartic acid, l-glutamic acid, l-malic acid, propionic acid, sucrose, Tween 40, *α*-d-glucose, *α*-d-lactose, and *α*-keto-butyric acid can be utilized as sole carbon sources in Biolog GEN III Micro-Plates. Acid is produced from esculin ferric citrate and potassium 5-ketogluconate. Cell wall contains alanine, glutamic acid, glycine and *meso*-diaminopimelic as diagnostic amino acids. The whole-cell hydrolysis contains arabinose, galactose, ribose and traces of glucose. The cellular polar lipid system includes DPG, PE, PG, PI, and PIM, as well as small amount of APL. The predominant respiratory quinone is MK-9(H_4_), with minor of MK-8(H_4_). The major fatty acids are C_18:1_*ω*9*c*, iso-C_16:0_, C_16:0_, iso-C_15:0_, and C_16:1_*ω*7*c*. The G+C content of the genomic DNA is 74.7%. The type strain is CPCC 205119^T^ (=I12A-02624^T^=NBRC 113528^T^=KCTC 49201^T^), isolated from the moss-dominated soil crust from the Shapotou NDER located in Tengger Desert situated at the Ningxia Hui Autonomous Region, the north of China. The assembly sequences for the draft genome of CPCC 205119^T^ has been deposited in GenBank with the accession number JAAGWK000000000.

## Discussion

Based on the genomic information, we summarized genetic characteristics of the novel species *M. deserti* sp. nov. accommodating three isolates from the desert microbiological soil crusts. The detailed phenotypic properties illustrated the abilities of these strains to adapt to environmental stress.

In core genome of the species *M. deserti*, 37 gene clusters (1.6%) were mapped to bacterial chemotaxis (ko02030, pathway number in KEGG Orthology) and flagellar assembly (ko02040) pathway which was related to cell motility (KO09142, KEGG Orthology number). These gene clusters, including chemotaxis family related genes *cheA, cheB*, *cheR*, *cheW*, *cheY*, *mcp*, *motA*, *motB*, *rbsB*, *tar*, and *tap*, flagellar basal-body rod protein coding genes *flgB* and *flgC*, flagellar basal-body rod modification protein coding gene *flgD*, flagellar hook protein coding genes *flgE* and *flgL*, flagellar biosynthesis protein coding genes *flhA*, *flhB*, *fliP*, *fliQ*, and *fliR*, RNA polymerase sigma factor for flagellar operon coding gene *fliA*, flagellin coding gene *fliC*, flagellar hook-basal body complex protein coding gene *fliE*, flagellar M-ring protein coding gene *fliF*, flagellar motor switch protein coding genes *fliG and fliY*, flagellum-specific ATP synthase gene *fliI*, flagellar motor switch protein coding gene *fliM*. These genes might encode some factors and proteins to help the cells to adapt to harsh environment by sensing chemical gradients in their habitats and then move toward more favorable conditions. The interaction between above factors and proteins caused a change in behavior, such as in direction or speed of rotation of flagella (Miller et al., 2009).

Phosphorus (P) acquisition and assimilation are of fundamental importance in cell physiology because P is a kind of required nutrient in many of the metabolic and energy-producing pathways in bacteria. The phosphorus sources include organic phosphorus and inorganic phosphorus. Bacteria transport inorganic phosphate by high affinity phosphate transport system PstSCAB coded by the *pstSCAB* operon (Hudek et al., 2016; Martín and Liras, 2021), PhnCDE transport system (coded by the *phnCDE* gene clusters; Hove-Jensen et al., 2010) and Pit transport systems (coded by *pitH* gene; Hoffer et al., 2001). Both transport systems are regulated by the two-component system PhoR-PhoP or PhoR-PhoB, and also interact with the phosphate modulator PhoU. To respond to phosphate limitation, some bacteria species may trigger the expression of genes encoding extracellular enzymes, including alkaline phosphatases PhoA (coded by *phoA* gene), PhoC (coded by *phoC* gene), and the phospholipase PhoD (coded by *phoD* gene; Apel et al., 2007; Moura et al., 2001). In the KAAS annotation results of the three strains, phosphate ABC transporters corresponding genes *pstS, pstA*, *pstB*, and *pstC*, phosphonate ABC transporters related genes *phnD*, *phnE* and *phnC*, two component system related genes phoR and phoD were all sorted.

The phenotypic assays showed that all the three strains could assimilate pyrophosphate, thiophosphate, dithiophosphate, phosphoenol pyruvate, 2-deoxy-d-glucose 6-phosphate, and cysteamine-S-phosphate, especially, they could solubilize tricalcium phosphate [Ca_3_(PO_4_)_2_] and phytin.

Assignment of metabolic pathways *via* the KEGG database, 2-deoxy-d-glucose-6-phosphate (C06369), dithiophosphate, phosphoenol pyruvate (C00074), and pyrophosphate (C00013) could be predicted from these three strains’ metabolic pathway. In the RAST annotation results (Figure 2), the phytase coding gene (*phyC*) was retrieved from all the three genomes. It could be proposed that phytase could catalyze the hydrolysis of inorganic orthophosphate from phytin (Kerovuo et al., 1998).

At the genomic level, alkaline phosphatase genes (*phoA, phD*) and phosphate transport-related genes (*phoU*, *phnC*, *phnD*, *phnE*, *phoB*, *phoP*, *phoR*, *pitH*, *pstA*, *pstB*, and *pstC*, and *pstS*), as well as inorganic pyrophosphatase coding gene (*ppa*), phytase coding gene (*phyC*), phosphate starvation-inducible protein coding gene (*phoH*), and a polyphosphate kinase coding gene (*ppk*) were retrieved from all these three genomes (Figure 2). Alkaline phosphatase A was a kind of nonspecific phosphate monoesterase. Alkaline phosphatase D with broad substrate specificity has phosphatase activity toward nucleotide and sugar phosphates (without phosphodiesterase activity; Gomez and Ingram, 1995).

All the genomes of these three strains contained one copy of *aglF* gene (*α*-glucoside transport system permease protein AglF coding gene), one copy of *aglG* gene (*α*-glucoside transport system permease protein AglG coding gene), one copy of *otsA* gene (trehalose-phosphate synthase gene), one copy of *otsB* gene (trehalose 6-phosphate phosphatase gene), two copies of *treS* genes (maltose alpha-d-glucosyltransferase gene), one copy of *treY* gene (maltooligosyl trehalose synthase gene), and one copy of *treZ* gene (malto-oligosyltrehalose trehalohydrolase gene). The proteins coded by these genes reported to be involved in the pathway of trehalose uptake and biosynthesis, which is part of starch and sucrose metabolism (ko00500), and these proteins were supposed to respond to the osmotic stress (Boos et al., 1990; Pan et al., 2008; Reina-Bueno et al., 2012; Chen et al., 2017; MacIntyre et al., 2020). Accumulated trehalose seems to have a major role in protecting the cells from osmotic pressure imbalance. As a major compatible solute, trehalose is involved in the osmotic stress response, cellular adaptation and survival to heat and desiccation stress (Reina-Bueno et al., 2012). The physiological tests results showed that strains CPCC 205119^T^, CPCC 205215, and CPCC 205251 could assimilate d-trehalose, which might contributed to maintaining the osmotic balance inside and outside the cell.

In the core genome of the species *M. deserti*, 96 gene clusters (4.2%) were mapped to replication and repair (KO09124) contained DNA replication (ko03030), base excision repair (ko03410), nucleotide excision repair (ko03420), mismatch repair (ko03430), homologous recombination (ko03440), and non-homologous end-joining (ko03450) pathway, such as *uvrA* (UvrABC system protein A coding gene), *uvrB* (UvrABC system protein B coding gene), *uvrC* (UvrABC system protein C coding gene), *uvrD* (DNA helicase II coding gene), *recA* (protein RecA coding gene), *recB* (RecBCD enzyme subunit RecB coding gene), *recC* (RecBCD enzyme subunit RecC coding gene), *recD* (RecBCD enzyme subunit RecD coding gene), *recF* (DNA replication and repair protein RecF coding gene), *recG* (ATP-dependent DNA helicase RecG coding gene), *recO* (DNA repair protein RecO coding gene), *recQ* (ATP-dependent DNA helicase RecQ coding gene), and *recR* (recombination protein RecR coding gene). Thirty-seven gene clusters (1.5%) were mapped to carbon fixation pathways in prokaryotes (ko00720), such as *coxS* (carbon monoxide dehydrogenase small chain coding gene), *coxM* (carbon monoxide dehydrogenase medium chain coding gene), and *coxL* (carbon monoxide dehydrogenase large chain coding gene). In the core genome, *katG* gene (catalase-peroxidase coding gene) was retrieved, and the protein coded by this gene could protect strains against toxic reactive oxygen species (ROS) including hydrogen peroxide as well as organic peroxides. The gene *csrA* was also retrieved from the core genome, this key regulator could bind to mRNA to regulate the gene expression, thereby, shift from rapid growth to stress survival, or might help the strain to survive in low carbon habitats by activating peptide uptake (Schultz and Matin, 1991; Lucchetti-Miganeh et al., 2008; Rasmussen et al., 2013).

These three strains of the species *M. deserti* showed a wide substrate-assimilation spectrum, including monosaccharide, oligosaccharide, polysaccharide (dextrin), glycerol, hexosephosphates, amino acids, carboxylic acids, fatty acids, and phosphate esters (Supplementary Table S3), which led to the assumption that these strains have evolved to enable access to various carbon sources or to be involved in catabolic pathways. Abundant and various of substrates that could be assimilated contain ester bonds. Own to the abundance of esterases (EC 3.1.1.X) in the strains, they could hydrolyze these ester bonds. So, these esterases might promote these strains survival abilities in dry environments (Bornscheuer, 2002).

Actinobacteria have been well recognized as the prolific producers of natural compounds (Hu et al., 2019; Yang et al., 2021). Typically, *Streptomyces* spp. and *Micromonospora* spp. are rich in secondary metabolite synthesis gene clusters (dozens; Carro et al., 2018; Kim et al., 2019). However, the results from antiSMASH database showed that in these three strains of the species *M. deserti*, only seven secondary metabolite gene clusters with moderate similarities to previously described secondary metabolite biosynthetic gene clusters were retrieved (Supplementary Table S5). These gene clusters exhibited 16–100% similarities to previously reported secondary metabolite biosynthetic gene clusters, such as 5-isoprenylindole-3-carboxylate *β*-d-glycosyl easter, alkyl-O-dihydrogeranyl-methoxyhydroquinones, arsono-polyketide, desferrioxamine, hiroshidine, isorenieratene and tallysomysin A gene clusters. While abundant stress-responding genes were found in the core genome of the newly established species, which were proposed to contribute greatly to their survival abilities in harsh desert environments. Therefore, the species *M. deserti* might serve as research model organisms for poineer microorganisms adaption and evolution in extreme environments.

## Data Availability Statement

The datasets presented in this study can be found in online repositories. The names of the repository/repositories and accession number(s) can be found in the article/Supplementary Material. The DDBJ/EMBL/GenBank accession numbers for the 16S rRNA gene sequences of strains CPCC 205119^T^, CPCC 205215 and CPCC 205251 are MK392028, MN567210 and MN567664, respectively. This whole genome shotgun projects of strains CPCC 205119^T^, CPCC 205215 and CPCC 205251 have been deposited at DDBJ/ENA/GenBank under the accession numbers JAAGWK000000000, WGGQ00000000 and JAABOZ000000000, respectively.

## Author Contributions

Z-MJ, B-HZ, and H-MS carried out the experiments and prepared the manuscript. TZ and L-YY collected the samples. Y-QZ designed the research and analyzed the data. All authors contributed to the article and approved the submitted version.

## Funding

This research was supported by National Natural Science Foundation of China (32170021 and 31670010), Beijing Natural Science Foundation (5212018), the National Infrastructure of Microbial Resources (NIMR-2021-3), and CAMS Innovation Fund for Medical Sciences (CIFMS; 2016-I2M-2-002).

## Conflict of Interest

The authors declare that the research was conducted in the absence of any commercial or financial relationships that could be construed as a potential conflict of interest.

## Publisher’s Note

All claims expressed in this article are solely those of the authors and do not necessarily represent those of their affiliated organizations, or those of the publisher, the editors and the reviewers. Any product that may be evaluated in this article, or claim that may be made by its manufacturer, is not guaranteed or endorsed by the publisher.

## Footnotes

^1^ https://lpsn.dsmz.de/genus/modestobacter

^2^ http://eztaxon-e.ezbiocloud.net

^3^ https://www.dsmz.de/collection/catalogue/details/culture/DSM-100206

^4^ http://www.cbcb.umd.edu/software/glimmer/

^5^ http://tandem.bu.edu/trf/trf.html

^6^ http://www5.esu.edu/cpsc/bioinfo/software/GIST/

^7^ http://phast.wishartlab.com/

^8^ https://www.uniprot.org/

^9^ https://www.ezbiocloud.net/tools/ani

^10^ https://ggdc.dsmz.de/

^11^ http://enve-omics.ce.gatech.edu/aai/

## Abbreviations

ANI: Average nucleotide identity
dDDH: Digital DNA–DNA hybridization
AAI: Amino acid identity
POCP: Percentage of conserved proteins
BSCs: Biological soil crusts
