# Supplementary material for: Properties of Modestobacter deserti sp. nov., a Kind of Novel Phosphate-Solubilizing Actinobacteria Inhabited in the Desert Biological Soil Crusts
Source: Front Microbiol. 2021 Nov 5;12:742798. doi: 10.3389/fmicb.2021.742798 (PMC8602919; doi:10.3389/fmicb.2021.742798)
Supplement: Supplementary file 1 [file Presentation_1.pdf]

Properties of *Modestobacter deserti* sp. nov., a kind of novel phosphate-solubilizing actinobacteria inhabited in the desert biological soil crust

Zhu-Ming Jiang[1], Bing-Huo Zhang[2], Hong-Min Sun[1], Tao Zhang[1], Li-Yan Yu[1], Yu-Qin Zhang[1*]

[1]Institute of Medicinal Biotechnology, Chinese Academy of Medical Sciences & Peking Union Medical College, Beijing 100050, P. R. China

[2]College of Life Science, Jiujiang University, Jiujiang 332005, PR China

Author for correspondence:

Yu-Qin Zhang

Tel: +86-10-83167110

Fax: +86-10-83167110

E-Mail: zhyuqin@126.com

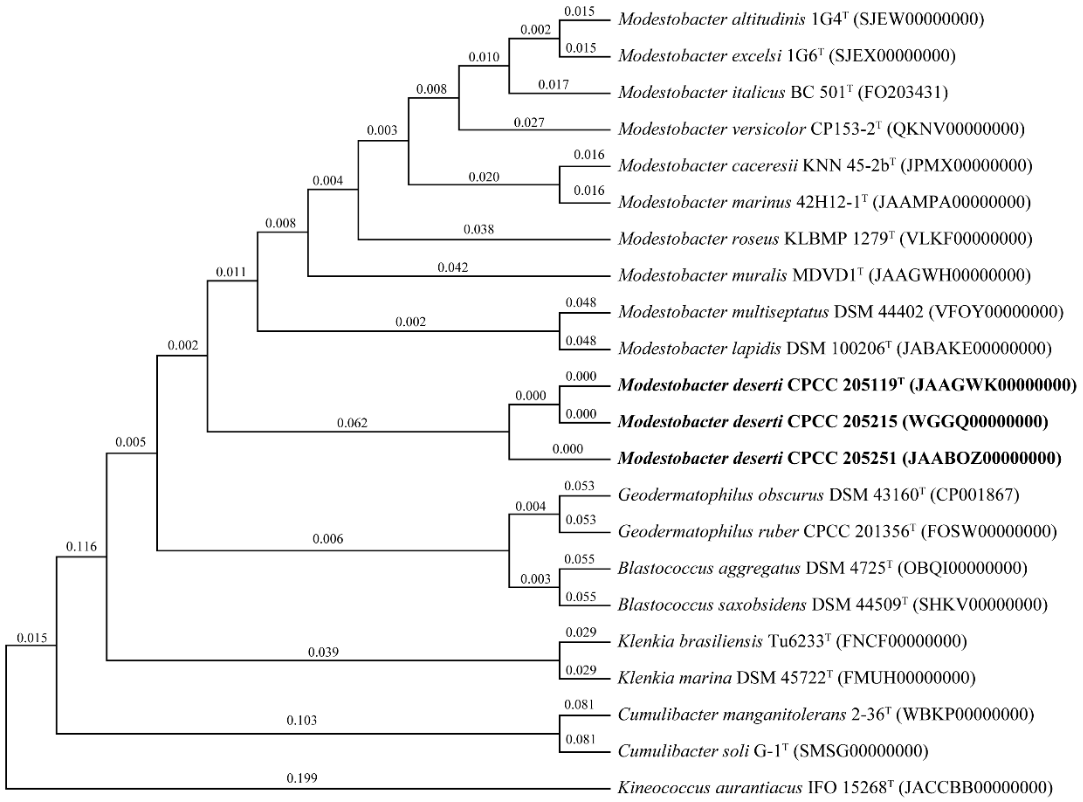

FIGURE S1 | Phylogenetic tree constructed by BPGA showing the relationship of the newly proposed species with other species of the family *Geodermatophilaceae* based on concatenated core genes.

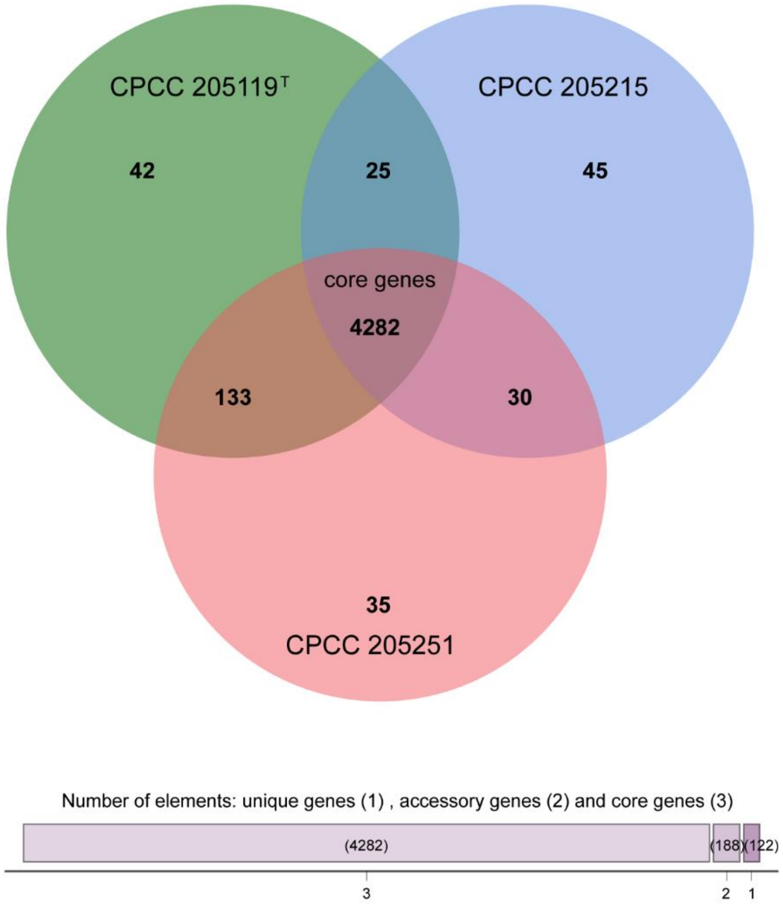

FIGURE S2 | Venn diagrams illustrating the number of core, accessory and unique genes among the pan genome of the species *Modestobacter deserti*.

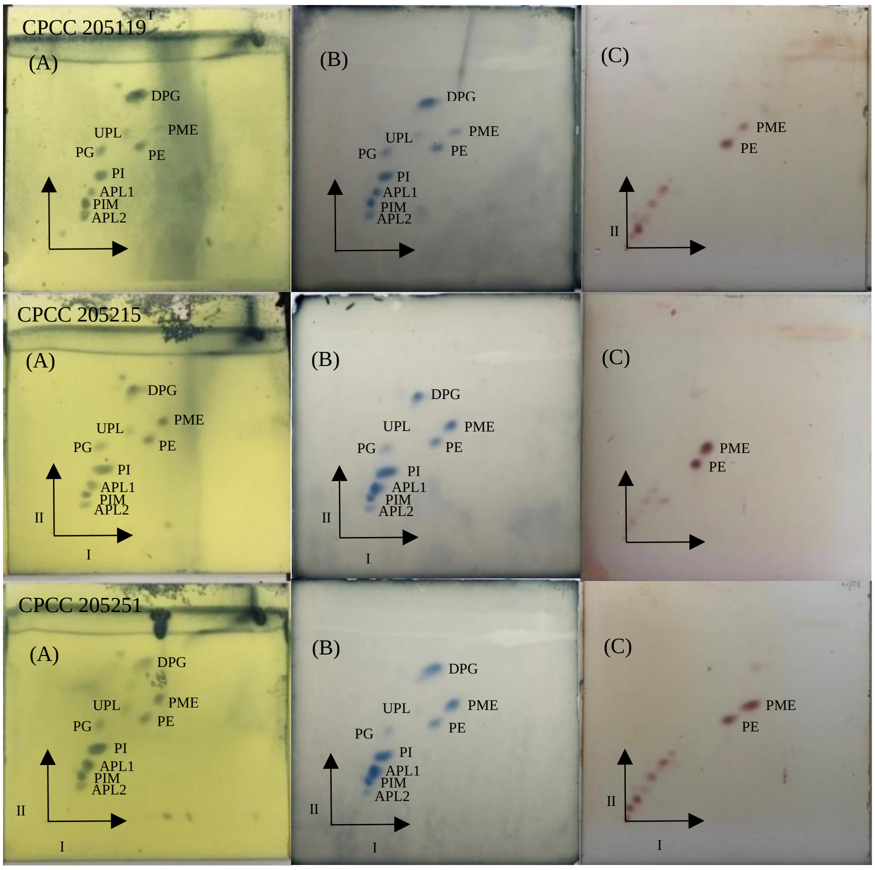

FIGURE S3 | Polar lipids profiles of the strain CPCC 205119^{T}, CPCC 205215 and CPCC 205251 separation by two-dimensional TLC. (A) was detected by spraying with molybdatophosphoric acid reagent; (B) was detected by spraying with phosphate stain reagent; (C) was detected by spraying with ninhydrin stain reagent. DPG, diphosphatidylglycerol; PE, phosphatidylethanolamine; PME, phosphatidylmethylethanolamine; PG, phosphatidylglycerol; PI, phosphatidylinositol; PIM, phosphatidylinositol mannosides; APL, aminophospholipid; UPL, unidentified phospholipid.

Table S1 Summary of genome features of strain CPCC 205119^T, CPCC 205215, CPCC 205251, *M. lapidis* DSM 100206^T and *M. multiseptatus* DSM 44402.

Features	*M. deserti* CPCC 205119^T	*M. deserti* CPCC 205215	*M. deserti* CPCC 205251	*M. lapidis* DSM 100206^T	*M. multiseptatus* DSM 44402
Genome size (Mbp)	4.84	4.84	4.92	4.90	6.09
G+C (%)	74.6	74.7	74.7	73.4	71.7
Contig	62	20	20	466	9
N50 Length (bp)	184,770	426,423	566,541	222,104	3,630,887
Genome coverage	470×	380×	380×	479×	265×
16S rRNA gene length (bp) retrieved from genome assembly	1,529	1,529	1,529	1,452	1,482
Genes (Total)	4,480	4,443	4,523	4,366	6,094
CDSs (Total)	4,424	4,385	4,465	4,291	6,038
Genes (RNA)	56	58	58	195	56
CDSs (protein)	4,323	4,257	4,361	4,096	5,651
rRNAs	5	7	7	11	6
tRNAs	48	48	48	87	47
Other ncRNA	3	3	3	97	3
Pseudo Genes	101	128	104	270	387
DDBJ/EMBL/GenBank accession number of darft genome	JAAGWK000000000	WGGQ00000000	JAABOZ000000000	JABAKE000000000	VFOY00000000
GenBank/RefSeq assembly accession number	GCA_010685995.1	GCA_009296285.1	GCA_010077965.1	GCA_019779905.1	GCA_006716365.1

Table S2 The pairwise values of dDDH, ANI and AAI and POCP among strains CPCC 205119^T, CPCC 205215 and CPCC 205251.

Note: ANI, values of average nucleotide identity; dDDH, digital DNA–DNA hybridization; AAI, average amino acid identity; POCP, percentage of conserved proteins. The values in parentheses are ANI, dDDH, AAI, and POCP from left to right

	Modestobacter deserti CPCC 205119^T	*Modestobacter deserti* CPCC 205215	*Modestobacter deserti* CPCC 205251
Modestobacter deserti CPCC 205119^T	(100.0, 100.0, 100.0, 100.0)	(100.0, 100.0, 100.0, 97.3)	(100.0, 100.0, 100.0, 98.5)
Modestobacter deserti CPCC 205215	(100.0, 100.0, 100.0, 97.3)	(100.0, 100.0, 100.0, 100.0)	(100.0, 100.0, 100.0, 97.4)
Modestobacter deserti CPCC 205251	(100.0, 100.0, 100.0, 98.5)	(100.0, 100.0, 100.0, 97.4)	(100.0, 100.0, 100.0, 100.0)

Table S3 Physiological and biochemical characteristics profiles of strain CPCC 205119^T, CPCC 205215 and CPCC 205251.

	CPCC 205119^T	CPCC 205215	CPCC 205251
**Assimilation of carbon sources (BIOLOG GEN III microplates):**			
Dextrin	+	+	+
D-maltose	+	+	+
D-trehalose	+	+	+
D-cellobiose	+	+	+
Gentiobiose	-	-	-
Sucrose	+	+	+
D-turanose	+	+	+
Stachyose	-	-	-
D-raffinose	-	-	-
α-D-lactose	+	+	+
D-melibiose	-	-	-
β-methyl-D-glucoside	-	-	-
D-salicin	+	+	+
N-acetyl-D-glucosamine	-	-	-
N-acetyl-β-D-mannosamine	-	-	-
N-acetyl-D-galactosamine	-	-	-
N-acetyl-neuraminic acid	-	-	-
α-D-glucose	+	+	+
D-mannose	+	+	+
D-fructose	+	+	+
D-galactose	+	+	+
3-methyl-glucose	-	-	-
D-fucose	-	-	-
L-fucose	-	-	-
L-rhamnose	-	-	-
Inosine	+	-	-
D-sorbitol	-	-	-
D-mannitol	-	-	-
D-arabitol	-	-	-
myo-inositol	+	-	+
Glycerol	+	+	+
D-glucose-6-PO_4	+	-	-
D-fructose-6-PO_4	+	-	-
D-aspartic acid	-	-	-
D-serine	-	-	-
Gelatin	+	+	+
Glycyl-L-proline	-	-	-
L-alanine	-	-	-

L-arginine	-	-	-
L-aspartic acid	+	+	+
L-glutamic acid	+	+	+
L-histidine	-	-	-
L-pyroglutamic acid	-	-	-
L-Serine	-	-	-
Pectin	-	+	+
D-galacturonic acid	-	-	-
L-galactonic acid lactone	-	-	-
D-gluconic acid	+	-	-
D-glucuronic acid	-	-	-
Glucuronamide	-	+	-
Mucic acid	-	-	-
Quinic acid	-	-	-
D-saccharic acid	-	-	-
p-hydroxy-phenylacetic acid	-	-	-
Methyl pyruvate	-	+	+
D-lactic acid methyl ester	-	-	-
L-lactic acid	-	-	-
Citric acid	-	-	-
α-keto-glutaric acid	-	-	-
D-malic acid	+	+	+
L-malic acid	+	+	+
Bromo-succinic acid	+	+	+
Tween 40	+	+	+
γ-amino-butryric Acid	-	-	-
α-hydroxy-butyric acid	-	+	+
β-hydroxy-D,L-butyric acid	-	-	-
α-keto-butyric acid	+	+	+
Acetoacetic acid	-	+	+
Propionic acid	+	+	+
Acetic acid	+	+	+
Formic acid	+	+	-
**Assimilation of nitrogen source (PM3B microplates):**			
Nitrite	-	-	-
Nitrate	-	-	+
L-asparagine	-	-	+
L-aspartic acid	+	-	-
L-cysteine	+	+	+
L-glutamic acid	-	-	+
L-glutamine	-	+	+
Glycine	-	-	+
L-histidine	-	-	-
L-isoleucine	-	+	+

L-lysine	-	-	-
L-methionine	-	-	-
L-proline	-	-	-
L-serine	-	-	-
L-tyrosine	+	+	+
L-valine	-	+	-
D-alanine	-	+	-
D-asparagine	-	-	+
D-glutamic acid	-	+	-
D-lysine	-	-	+
D-serine	-	-	+
D-valine	-	-	-
L-citrulline	-	-	-
L-ornithine	-	-	-
N-acetyl-D,L-glutamic acid	+	+	-
γ-amino-N-butyric acid	-	+	-
ε-amino-N-caproic acid	-	+	-
D,L-α-amino-caprylic acid	-	+	+
Methylamine	-	+	-
N-amylamine	-	-	-
N-butylamine	-	-	-
Ethylamine	-	+	-
Ethylenediamine	-	+	-
Putrescine	-	+	-
Tyramine	-	-	-
Acetamide	-	+	-
Formamide	-	-	-
Glucuronamide	-	-	-
D-glucosamine	-	+	-
D-galactosamine	+	+	+
D-mannosamine	+	+	+
N-acetyl-D-glucosamine	-	-	-
N-acetyl-D-mannosamine	-	-	-
Adenine	-	-	-
Adenosine	-	+	-
Guanosine	-	+	-
Xanthine	+	+	+
Xanthosine	-	+	+
Cytidine	-	+	-
Uric acid	+	+	+
Alloxan	-	+	-
Allantoin	+	+	+
Ala-Asp	+	-	+
Ala-Gln	+	+	+

Ala-Glu	+	+	-
Ala-Gly	-	+	-
Ala-Leu	-	+	-
Ala-Thr	-	+	-
Gly-Asn	-	+	+
Gly-Gln	-	+	+
Gly-Glu	-	+	-
**Assimilation of phosphorus source (PM4A Microplates):**			
Pyrophosphate	+	+	+
Trimetaphosphate	-	-	+
Tripolyphosphate	-	-	+
Hypophosphite	-	-	+
Thiophosphate	+	+	+
Dithiophosphate	+	+	+
Adenosine-3'-monophosphate	+	-	-
Adenosine-5'-monophosphate	+	-	-
Adenosine-3',5'-cyclic monophosphate	-	-	+
Guanosine-2'-monophosphate	-	-	+
Guanosine-3'-monophosphate	-	-	-
Guanosine-3',5'-cyclic monophosphate	-	-	-
Cytidine-2',3'-cyclic monophosphate	-	-	-
Uridine-2'-monophosphate	-	-	+
D,L-α-glycerol phosphate	+	-	+
D-3-phospho-glyceric acid	-	-	+
Phosphoenol pyruvate	+	+	+
Phospho-glycolic acid	-	-	+
Phosphoryl choline	-	-	-
Methylene diphosphonic acid	-	+	+
2-deoxy-D-glucose-6-phosphate	+	+	+
D-glucosamine-6-phosphate	-	-	-
D-mannose-1-phosphate	-	+	+
D-mannose-6-phosphate	-	-	+
Cysteamine-S-phosphate	+	+	+
Phosphocreatine	-	-	-
**Assimilation of Sulfur source (PM4A Microplates):**			
N-acetyl-L-cysteine	+	-	+
Glutathione	-	-	-
L-methionine sulfone	-	+	-
L-djenkolic acid	+	-	+
1-thio-β-D-glucose	-	-	-
Butane sulfonic acid	+	-	-
2-hydroxyethane sulfonic acid	+	-	-
Methane sulfonic acid	+	-	-
**Enzyme activities (API-ZYM tests):**			

Alkaline phosphatase	+	+	+
Esterase(C4)	+	+	+
Esterase lipase(C8)	+	+	+
Lipase (C14)	+	+	+
Leucine arylamidase	+	+	+
Valine arylamidase	+	+	+
Cystine arylamidase	+	+	+
Trypsin	+	+	+
α-chymotrypsin	+	+	+
Acid phosphatase	+	+	+
Naphthol-AS-B1-phosphohydrolase	+	+	+
α-galactosidase	+	+	+
β-galactosidase	+	+	+
β-glucuronidase	-	-	-
α-glucosidase	+	+	+
β-glucosidase	+	+	+
N-acetyl-*β*-glucosaminidase	-	-	-
α-mannosidase	-	-	-
α-fucosidase	-	-	-
Argineine dihydrolase	+	+	+
Urease	+	+	+
**Acid production from (API 50 CH):**			
Esculin ferric citrate	+	+	+
Potassium 5-ketogluconate	+	+	+

Table S4. Fatty acids profile of strains CPCC 205119^T, CPCC 205215 and CPCC 205251.

TR, trace amount (<0.5 %); ND, not detected

Fatty acids (%)	CPCC 205119^T	CPCC 205215	CPCC 205251
**Saturated fatty acids**			
$C_{14:0}$	2.3	1.6	1.6
$C_{15:0}$	0.9	0.7	1.0
$C_{16:0}$	11.0	6.7	7.9
$C_{17:0}$	1.6	1.5	2.2
$C_{18:0}$	5.8	4.3	5.3
$C_{19:0}$	ND	ND	2.2
$C_{20:0}$	ND	ND	3.1
**iso-branched fatty acid**			
Iso- $C_{14:0}$	0.6	0.9	0.9
Iso- $C_{15:0}$	8.0	7.2	6.1
Iso- $C_{16:0}$	16.1	20.9	11.2
Iso- $C_{17:0}$	1.1	1.8	0.9
**anteiso-branched fatty acid**			
anteiso- $C_{15:0}$	0.8	TR	1.3
anteiso- $C_{17:0}$	1.0	1.1	0.9
**Unsaturated fatty acids**			
$C_{15:1}$ B	TR	TR	0.9
Iso-$C_{16:1}$ H	0.6	1.1	2.3
$C_{16:1}$ $\omega 7c$	6.6	7.2	9.0
$C_{17:1}$ $\omega 8c$	4.2	7.3	11.8
$C_{18:1}$ $\omega 9c$	29.7	28.1	18.9
**Hydroxy fatty acids**			
Iso-$C_{16:0}$ 2-OH	4.2	2.9	2.5
Iso-$C_{17:0}$ -OH	ND	TR	0.7
$C_{16:0}$ 2-OH	0.9	0.6	1.0

Table S5. Secondary metabolite biosynthesis gene clusters predicted from the of 3 strains' genomes *of* the species *Modestobacter deserti*.

Genomic location	Type	Secondary metabolite synthesis gene cluster	Smilarity
**CPCC 205119^T**			
Scaffold1_8	linardin/T3PKS(Linaridin cluster/Type III PKS)	alkyl-O-dihydrogeranyl-methoxyhydroquinones	28 %
Scaffold2_1	terpene	isorenieratene	25 %
Scaffold3_1	siderophore	desferrioxamine	100 %
Scaffold4_1	terpene	arsono-polyketide	16 %
Scaffold4_1	NRPS	tallysomysin A	27 %
Scaffold5_1	T2PKS(Type II PKS)	hiroshidine	24 %
Scaffold9_1	NRPS-like	5-isoprenylindole-3-carboxylate *β*-D-glycosyl easter	19 %
**CPCC 205215**			
NODE_1	NRPS	tallysomysin A	27 %
NODE_1	terpene	arsono-polyketide	16 %
NODE_1	T2PKS	hiroshidine	24 %
NODE_2	terpene	isorenieratene	25 %
NODE_4	siderophore	desferrioxamine E	100 %
NODE_5	linardin/T3PKS	alkyl-O-dihydrogeranyl-methoxyhydroquinones	28 %
NODE_7	NRPS-like	5-isoprenylindole-3-carboxylate *β*-D-glycosyl easter	19 %
**CPCC 205251**			
Scaffold2_1	siderophore	desferrioxamine E	100 %
Scaffold3_3	terpene	isorenieratene	25 %

Scaffold4_1	terpene	arsono-polyketide	16 %
Scaffold4_1	NRPS	tallysomysin A	27 %
Scaffold6_2	T2PKS	hiroshidine	24 %
Scaffold9_1	linardin/T3PKS	alkyl-O-dihydrogeranyl-methoxyhydroquinones	28 %
Scaffold9_3	ranthipeptide	/	/
Scaffold11_1	NRPS-like	5-isoprenylindole-3-carboxylate *β*-D-glycosyl easter	19 %
